# Fragmented Healthcare Experiences of Older Asian Americans Living with HIV

**DOI:** 10.1093/geroni/igaf122.2679

**Published:** 2025-12-31

**Authors:** Jen-Hao Chen, Cheng-Shi Shiu, Wei-Ti Chen

**Affiliations:** National Chengchi University, Taipei, Taiwan (Republic of China); National Taiwan University, Taipei, Taiwan (Republic of China); University of California, Los Angeles, Los Angeles, California, United States

## Abstract

As the Asian American population with HIV ages, their healthcare experiences remain understudied. Understanding their challenges is critical for improving geriatric HIV care and informing culturally competent interventions for Asian American communities. We conducted qualitative interviews with 34 HIV-positive Asian American older adults in Los Angeles between 2018 and 2019. Analysis focused on identified patterns related to healthcare discontinuities, provider-patient interactions, and system navigation difficulties. Participants experienced significant healthcare fragmentation, including frequent provider changes, service disruptions, and inefficient care coordination. Many patients switched providers due to insurance changes, discrimination concerns, or communication barriers, disrupting treatment continuity. Limited English proficiency further complicated care access, as many patients relied on family members for translation, leading to misinterpretations, incomplete medical discussions, and a loss of autonomy due to the absence of professional interpreters in HIV clinics. Stigma and medical mistrust also contributed to delayed treatment, with patients avoiding care to prevent disclosure within both healthcare settings and their own communities, increasing their reliance on emergency care. Fragmentation between HIV treatment, general healthcare, and mental health services led to inconsistent medication management and poor psychosocial support. Additionally, many participants expressed concerns about discrimination in long-term care facilities, discouraging them from seeking aging-related healthcare planning. Findings highlight the need for culturally competent geriatric HIV care, including professional interpretation services, improved care coordination, and community-based aging-HIV programs to ensure equitable access and improved health outcomes.

